# SmSPL6 Induces Phenolic Acid Biosynthesis and Affects Root Development in *Salvia miltiorrhiza*

**DOI:** 10.3390/ijms22157895

**Published:** 2021-07-23

**Authors:** Yao Cao, Rui Chen, Wen-Tao Wang, Dong-Hao Wang, Xiao-Yan Cao

**Affiliations:** Key Laboratory of the Ministry of Education for Medicinal Resources and Natural Pharmaceutical Chemistry, National Engineering Laboratory for Resource Development of Endangered Crude Drugs in Northwest of China, Shaanxi Normal University, Xi’an 710062, China; caoyao@snnu.edu.cn (Y.C.); chenruirui@snnu.edu.cn (R.C.); wangwentao@snnu.edu.cn (W.-T.W.)

**Keywords:** root development, *salvia miltiorrhiza*, salvianolic acid B, SPL, transcription factor

## Abstract

*Salvia miltiorrhiza* is a renowned model medicinal plant species for which 15 *SQUAMOSA PROMOTER BINDING PROTEIN-LIKE (SPL)* family genes have been identified; however, the specific functions of *SmSPLs* have not been well characterized as of yet. For this study, the expression patterns of *SmSPL6* were determined through its responses to treatments of exogenous hormones, including indole acetic acid (IAA), gibberellic acid (GA_3_), methyl jasmonic acid (MeJA), and abscisic acid (ABA). To characterize its functionality, we obtained *SmSPL6-*ovexpressed transgenic *S. miltiorrhiza* plants and found that overexpressed *SmSPL6* promoted the accumulation of phenolic acids and repressed the biosynthesis of anthocyanin. Meanwhile, the root lengths of the *SmSPL6*-overexpressed lines were significantly longer than the control; however, both the fresh weights and lateral root numbers decreased. Further investigations indicated that *Sm*SPL6 regulated the biosynthesis of phenolic acid by directly binding to the promoter regions of the enzyme genes *Sm4CL9* and *SmCYP98A14* and activated their expression. We concluded that *Sm*SPL6 regulates not only the biosynthesis of phenolic acids, but also the development of roots in *S. miltiorrhiza*.

## 1. Introduction

*Salvia miltiorrhiza* Bunge, which belongs to the Labiatae family, is a significant medicinal plant [1]. Its dried roots (referred to as Danshen in Chinese), in combination with other herbs, have been used extensively for many years to treat various conditions [2], including cardiovascular diseases [3,4], menstrual disorders [5], inflammation prevention [6], hepatocirrhosis [7], as an anti-osteoporotic [2], and so on. Many capsules, dripping pills, injection solutions, and tablets used in clinical applications are comprised of Danshen. *S. miltiorrhiza* contains diverse chemical components, encompassing approximately 50 diterpenoid quinones, more than 30 hydrophilic phenolic acids, and several essential oil constituents. According to pharmacological investigations, lipid-soluble tanshinones and water-soluble phenolic acids are the main active ingredients of *S. miltiorrhiza* [1,8].

The biological activities of lipid-soluble tanshinones, such as tanshinone and tanshinol A, include cardio-cerebrovascular protection and serve as anticancer and anti-inflammatory agents [9]. Hydrophilic phenolic acids, such as salvianolic acid B (SalB) and rosmarinic acid (RA), have potent anti-oxidative, anti-coagulation, and anti-inflammatory properties [10]. As the main active ingredient of phenolic acids, SalB is designated as a primary component of Danshen in the official Chinese Pharmacopoeia [11]. In *S. miltiorrhiza*, the biosynthesis of RA and SalB originates from phenylpropanoid and tyrosine-derived pathways [12]. The phenylpropanoid pathway includes three key enzymes, namely phenylalanine ammonia lyase (PAL), cinnamate 4-hydroxylase (C4H), and 4-coumarate-CoA ligase (4CL). For the tyrosine-derived pathway, 4-hydroxyphenylpyruvate reductase (HPPR) and tyrosine aminotransferase (TAT) are the key enzymes. Furthermore, rosmarinic acid synthase (RAS) and cytochrome P450 monooxygenase C3′H (CYP98A14) catalyze the biosynthesis of RA and SalB [13,14,15]. Many reports have indicated that several transcription factors participate in the regulation of phenolic acid accumulation in *S. miltiorrhiza*. For instance, *Sm*bHLH3 is a negative regulatory factor in the biosynthesis of phenolic acids [16]. In contrast, the expression of *Sm*MYB111 can positively regulate the accumulation of phenolic acids [17].

The *SQUAMOSA PROMOTER BINDING PROTEIN-LIKE* (*SPL*) transcription factor family is characterized by a highly conserved SBP-box domain, which plays important roles in plant growth and development [18]. The SBP-box domain consists of 76 amino acids that contain two zinc finger sites, which specifically bind to the GTAC core motif [19]. *SPL* genes have been widely investigated in many plants. There are 16 *SPL* genes in *Arabidopsis thaliana* [19], 18 *SPL* genes in *Betula luminifera* [20], 19 *SPLs* in rice [21], and 15 *SPLs* have been identified for *S. miltiorrhiza* [22].

In *Arabidopsis*, the functions of *SPL* genes have been thoroughly investigated. Most *SPLs* are miR156 targets, which is conserved and age-regulated in microRNA. The miR156-SPL regulatory module controls multiple developmental processes, including the juvenile-to-adult phase transition [23], flower formation [24], and root development [25,26,27]. It has been reported that *At*SPL9 and *At*SPL15 mediate lateral bud growth and branching [28]. *At*SPL9 might interact with DELLA proteins (GA signaling pathway repressors) to promote the initiation of *Arabidopsis* axillary buds [29,30]. *At*SPL9 also interacts with JAZ proteins and contributes to insect resistance in young plants [31], whereas *At*SPL9 is involved in controlling the innate immunity of *A. thaliana* [32]. Furthermore, *At*SPL9 prevents the expression of anthocyanin biosynthetic genes from down-regulating anthocyanin accumulation by directly interfering with the formation of a MYB-bHLH-WD40 transcriptional complex [33].

Although 15 members of the *SPL* family have been identified in *S. miltiorrhiza* [22], none of these have been functionally experimentally characterized to date. According to phylogenetic tree analysis, *S.* *miltiorrhiza SPL6* (*SmSPL6*) and *AtSPL9* tend to cluster in the same subgroup [22]. We speculated as to whether *Sm*SPL6 might be involved in the accumulation of active ingredients in *S.* *miltiorrhiza*. To investigate the functionality of *SmSPL6*, we characterized its expression patterns and gain-of-function phenotype. Here, we found that *SmSPL6* responded to treatments with the exogenous hormones indole acetic acid (IAA), gibberellic acid (GA_3_), methyl jasmonic acid (MeJA), and abscisic acid (ABA). The overexpression of *SmSPL6* promoted the accumulation of RA and SalB by directly binding to the promoter regions of *SmCYP98A14* and *Sm4CL9* and activating their expression. Meanwhile, *Sm*SPL6 repressed the biosynthesis of anthocyanins and altered the phenotype of root systems. All of the results indicated that *Sm*SPL6 is a strong regulator of both secondary metabolites and root development.

## 2. Results

### 2.1. Expression Patterns of SmSPL6 in S. miltiorrhiza

**Figure 1:**
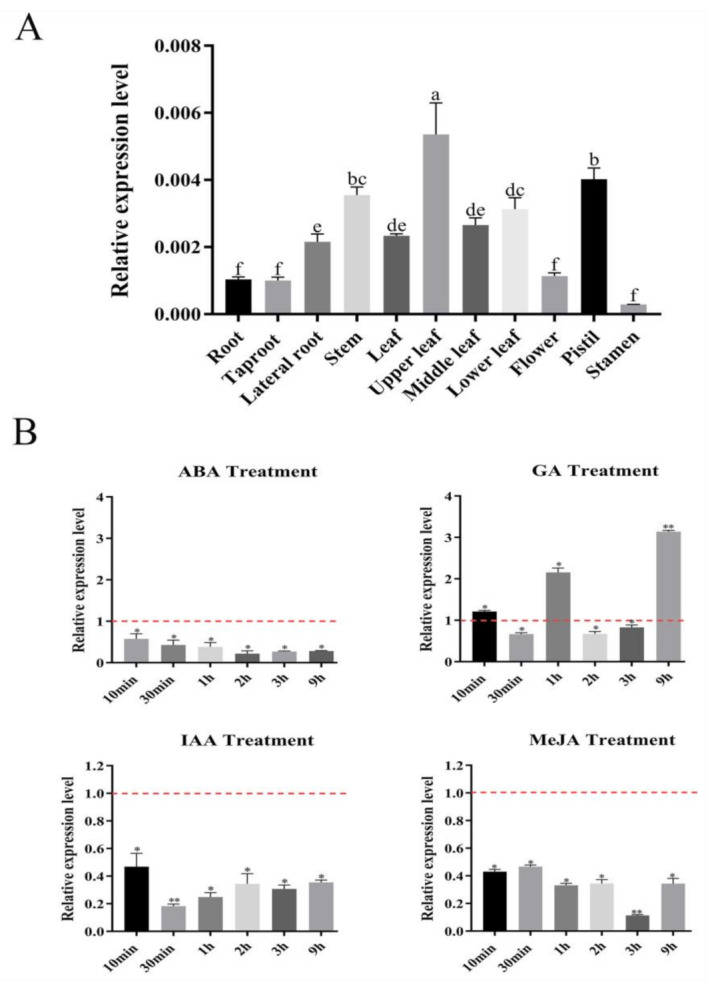
Expression profiles of *SmSPL6* in *Salvia miltiorrhiza*. (**A**) The expression of *SmSPL6* in different tissues. (**B**) Expression changes in response to treatment with 5 mM MeJA, 0.1 mM ABA, 0.1 mM IAA, and 0.1 mM GA_3_. All data are means of three biological replicates, with error bars indicating SD, red dotted line indicates the control which was set to 1. One-way ANOVA (followed by Tukey’s comparisons) tested for significant differences between means (indicated by different letters at *p* < 0.05). * and ** represent a significant difference at *p* < 0.05 and *p* < 0.01 compared with the control, respectively.

To investigate the expression patterns of *SmSPL6*, we extracted RNA from different tissues of 2-year-old *S. miltiorrhiza* and converted the RNA into cDNA for quantitative reverse transcription PCR (qRT-PCR) analysis. The results indicated that *SmSPL6* was expressed in all detected tissues of *S. miltiorrhiza*, with the highest expression level in the upper leaves (Figure 1A). We analyzed the promoter fragment of *SmSPL6* by PlantCARE and found cis-elements in response to IAA, GA_3_, and ABA (Table 1). In addition, the MeJA was also used to treat the *S. miltiorrhiza* plantlets. The results of qRT-PCR revealed that *SmSPL6* responded to IAA, GA_3_, MeJA, and ABA treatments. Exogenous IAA, ABA, or MeJA treatments significantly repressed the expression of *SmSPL6* (Figure 1B).

**Figure 2:**
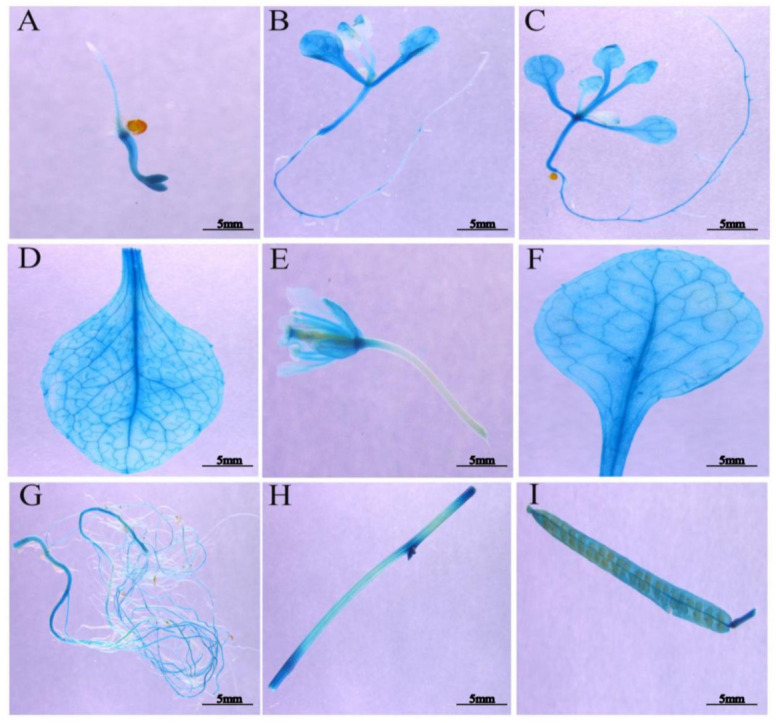
Temporal and spatial expression patterns of *SmSPL6*. (**A**) Two days after germination. (**B**) Seven days after germination. (**C**) Ten days after germination. (**D**) Rosette leaf. (**E**) Flower. (**F**) Stem leaf. (**G**) Root at the flowering stage. (**H**) Stem. (**I**) Silique.

To further examine the spatial expression patterns of *SmSPL6*, we constructed the 862 bp promoter region of *SmSPL6* into the pCAMBIA1391z to generate *ProSmSPL6::GUS* transgenic *Arabidopsis* plants and performed GUS histochemical staining. We observed a strong GUS signal for both the reproductive period and vegetative phase of *Arabidopsis* (Figure 2). However, no GUS signals were observed in the root tips (Figure 2A–C) or newly formed lateral roots (Figure 2B).

### 2.2. SmSPL6 Is Located in the Nucleus and Is Involved in Transcriptional Activation

**Figure 3:**
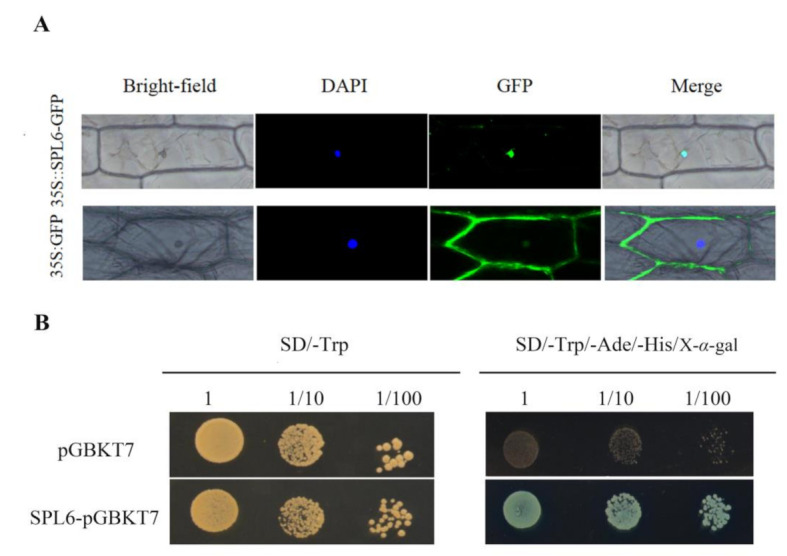
Subcellular location and transcription activity analysis of *Sm*SPL6 protein. (**A**) Subcellular location of *Sm*SPL6. The fluorescence signal was observed via laser scanning confocal microscope. DAPI, 4′, 6-diamidino-2-phenylindole is a blue fluorescent DNA stain that was used for indicating nucleus region. (**B**) Transcription activity analysis of *Sm*SPL6 protein. Yeast colonies with three different dilutions were grown on the SD/-Trp medium, then spotted on the SD/-Trp/-Ade/-His/X-α-gal. X-α-gal: 5-Bromo-4-chloro-3-indolyl-α-D-galactoside medium, the color reaction substrate of α-galactosidase.

To determine the subcellular localization of *Sm*SPL6, the open reading frame (ORF) of *SmSPL6* was cloned and fused to a green fluorescent protein (GFP) reporter gene, driven by the *35S* promoter of the cauliflower mosaic virus. *35Spro::SmSPL6*-GFP was transiently expressed in the onion epidermis and *35Spro*::GFP was employed as a positive control. The GFP signal indicated that the *Sm*SPL6 was specifically localized within the nucleus (Figure 3A), which was consistent with our expectation.

Furthermore, the recombinant vector pGBKT7-*SmSPL6* was transformed into the yeast strain AH109 for analyzing the transcriptional activation activities of *Sm*SPL6. Yeast containing the pGBKT7-*SmSPL6* or the negative control plasmid pGBKT7 grew well on the SD/-Trp solid medium; however, only the former grew normally on the SD/-Trp/-His/-Ade solid medium with X-α-gal and turned blue (Figure 3B). These results indicated that *Sm*SPL6 has transcriptional activation activity, and that *Sm*SPL6 is a functional transcription factor.

### 2.3. Generation of SmSPL6-Overexpressed Transgenic S. miltiorrhiza

**Figure 4:**
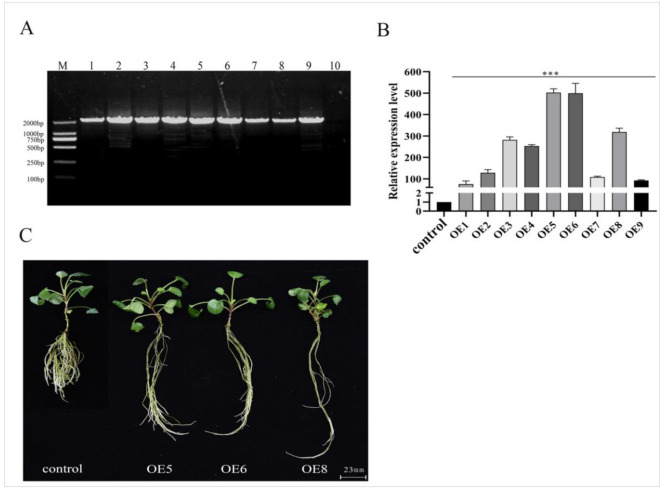
Identification and phenotype of the *SmSPL6*-overexpressed transgenic *Salvia miltiorrhiza*. (**A**) PCR-amplification product from the genome DNA of transgenic lines and the control. Forward primer: *CaMV35S*–F, reverse primer: *SmSPL6*–R, full length: 1833 bp. Lanes M, DL2000 DNA marker; 1–9, different transgenic lines; 10, control line obtained by plant tissue culture without *Agrobacterium* infection. (**B**) qRT-PCR analysis. Fold changes reflect the expressions of transgenic lines compared with the control expression, where the control values were set to 1. All data are the means of three replicates, with error bars indicating SD; *** represents a significant difference at *p* < 0.01 in contrast to the control. (**C**) Phenotype of the *SmSPL6*-overexpressed OE5, OE6, and OE8 transgenic lines and the control.

To characterize the functionality of the *SmSPL6* in *S. miltiorrhiza*, *SmSPL6*-overexpressed (OE) transgenic *S. miltiorrhiza* plants were obtained via the EHA105-mediated leaf disc transformation method. Nine independent *SmSPL6*-OE lines were verified through the presence of a sequence of 1833 bp that contained a *CaMV 35S* promoter and *SmSPL6* (Figure 4A). The *SmSPL6* expression levels in the control and *SmSPL6*-OE lines were analyzed (Figure 4B). We observed that OE5, OE6, and OE8 showed higher expression levels than the other OE lines; thus, they were selected for further analysis.

Subsequently, we observed the root phenotypes of 2-month-old *SmSPL6*-OE lines and a control. The lateral root numbers and fresh weight of the OE5, OE6, and OE8 transgenic lines were decreased; however, the lengths and diameters were increased in contrast to the control. These results revealed that the overexpression of *SmSPL6* affected the root phenotype of *S. miltiorrhiza* (Figure 4C, Table 2).

### 2.4. SmSPL6 Represses the Accumulation of Anthocyanin

**Figure 5:**
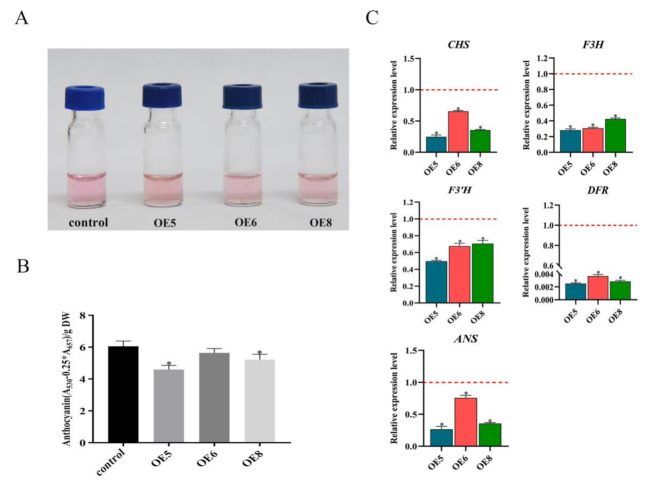
*Sm*SPL6 negatively regulates the biosynthesis of anthocyanin. (**A**) Anthocyanin extracted from the whole plant of the transgenic lines and the control. (**B**) Concentrations of anthocyanin in the transgenic lines and the control. (**C**) Expression changes of the enzyme genes for the biosynthetic pathway of anthocyanin. *CHALCONE SYNTHASE* (*CHS*), *FLAVANONE 3-HYDROXYLASE* (*F3H*), *FLAVONOID 3′-HYDROXYLASE* (*F3’H*), *DIHYDROFLAVONOL REDUCTASE* (*DFR*), *ANTHOCYANIDIN SYNTHASE* (*ANS*). All data are the means of three biological replicates, with error bars indicating SD. Red dotted lines indicate the control value, which was set to 1. * represents a significant difference at *p* < 0.05 compared with the control.

Since phylogenetic tree analysis indicated that *SmSPL6* tended to cluster with *AtSPL9* [22], which involves anthocyanin accumulation [33], we quantified the content of anthocyanin in the *SmSPL6*-OE lines and the control (Figure 5A). As anticipated, the content of total anthocyanin in the *SmSPL6*-OE lines (OE5, OE6, and OE8) was lower than that of the control (Figure 5B). Among the three transgenic lines, OE5 and OE8 showed significant differences. The results indicated that *Sm*SPL6 repressed the accumulation of anthocyanin in *S. miltiorrhiza*.

We further determined the expression levels of the key enzyme genes *CHALCONE SYNTHASE* (*CHS*), *FLAVANONE 3-HYDROXYLASE* (*F3H*), *FLAVONOID 3′-HYDROXYLASE* (*F3′H*), *ANTHOCYANIDIN SYNTHASE* (*ANS*), and *DIHYDROFLAVONOL REDUCTASE* (*DFR*) of the anthocyanin biosynthetic pathway in the transgenic lines, with the results showing that overexpressed *Sm*SPL6 could inhibit their transcription (Figure 5C). 

### 2.5. SmSPL6 Positively Regulates the Biosynthesis of RA and SalB

**Figure 6:**
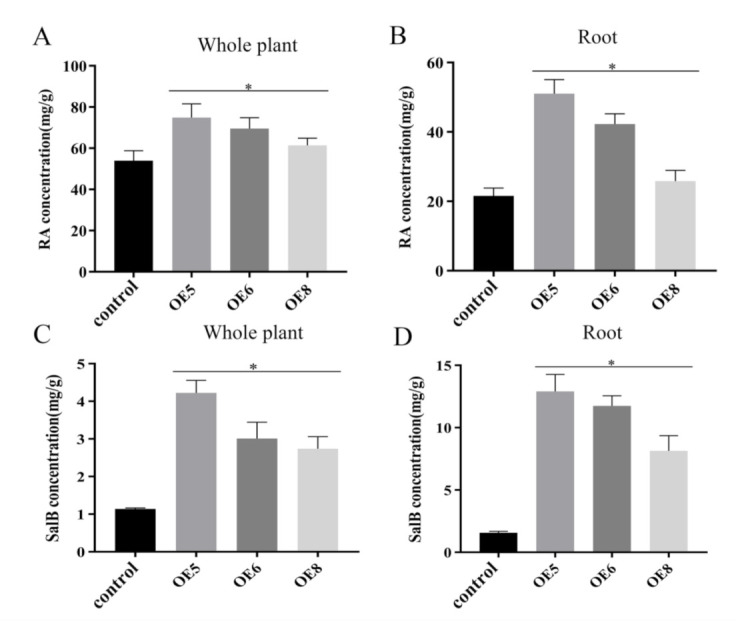
Concentrations of salvianolic acid B (SalB) in the whole plant (**C**) or the roots (**D**) and rosmarinic acid (RA) in the whole plant (**A**) or the roots (**B**) of the *SmSPL6*-overexpressed (OE) transgenic lines and the control. All data are the means of three biological replicates, with error bars indicating SD; * represents a significant difference at *p* < 0.05 compared with the control.

RA and SalB are the main active ingredients of *S. miltiorrhiza*. We determined the contents of RA and SalB using the HPLC method in the *SmSPL6*-OE lines (OE5, OE6, and OE8). The results revealed that the contents of RA and SalB were significantly increased, both in the whole plantlets and in the roots of the *SmSPL6*-OE lines, among which the OE5 line had the largest fold change (Figure 6). The contents of RA in the whole plantlets of OE5, OE6, and OE8 were 1.39, 1.29, and 1.14 times that of the control, respectively, and those of SalB were 3.69, 2.64, and 2.39 times that of the control, respectively. In the roots, the fold changes of RA in OE5, OE6, and OE8 were 2.36, 1.96, and 1.21 times that of the control, respectively, and those of SalB were 8.19, 7.47, and 5.18 times, respectively. These results indicated that *Sm*SPL6 is a positive regulator for the biosynthesis of RA and SalB, particularly in the roots.

**Figure 7:**
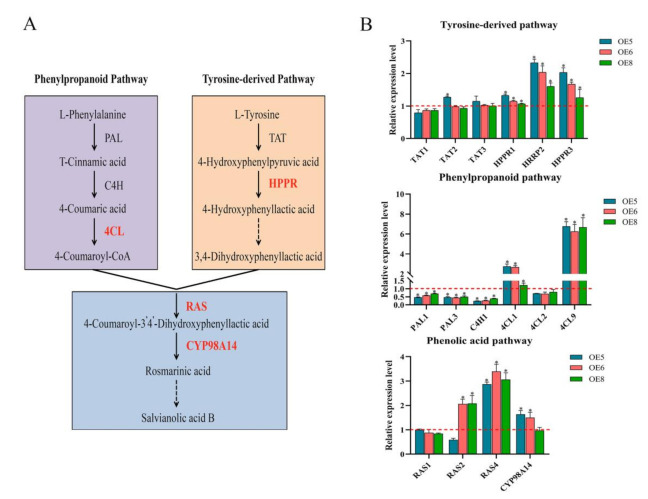
Expression changes of enzyme genes for the phenolic acid biosynthetic pathway in the *SmSPL6*-overexpressed (OE) transgenic lines. (**A**) Proposed biosynthetic pathway for phenolic acids (red indicates genes activated by *Sm*SPL6). TAT, tyrosine aminotransferase; HPPR, hydroxyl phenylpyruvate reductase; PAL, phenylalanine ammonia lyase; C4H, cinnamate 4-hydroxylase; 4CL, hydroxycinnamate-CoA ligase; RAS, rosmarinic acid synthase; and CYP, cytochrome P450 enzymes. (**B**) Expression changes of enzyme genes for the tyrosine pathway, phenylpropanoid pathway, and specific phenolic acid pathway in the *SmSPL6*-OE lines. The expression level in the control was set to 1 (shown as red dotted lines). All data are the means of three biological replicates, with error bars indicating SD; * represents a significant difference at *p* < 0.05 compared with the control.

We detected the expression levels of enzyme genes involved in the phenolic acid biosynthesis pathway in the roots (Figure 7A). Our qRT-PCR results indicated that most of these genes, including *SmHPPR1*, *SmHPPR2*, *SmHPPR3*, *Sm4CL1*, *Sm4CL9*, *SmRAS2*, *SmRAS4*, and *SmCYP98A14*, were significantly up-regulated (Figure 7B), particularly the expression level of *Sm4CL9*, which showed the largest fold change in every OE line.

### 2.6. SmSPL6 Binds Directly to the Promoter of SmCYP98A14 and Sm4CL9

**Figure 8:**
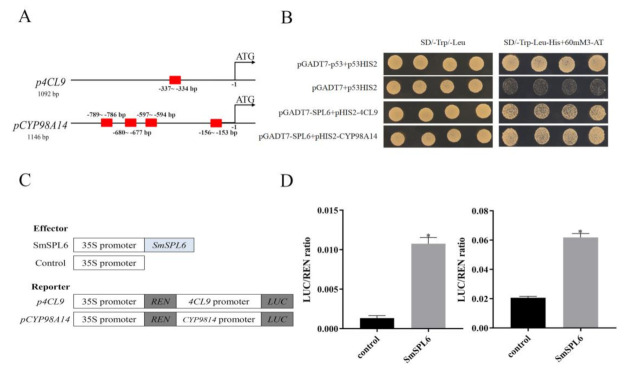
*Sm*SPL6 binds to the promoter regions of *Sm4CL9* and *SmCYP98A14* and activates their expression. (**A**) GTAC motifs in the promoter regions of *Sm4CL9* and *SmCYP98A14*. Red rectangles represent the GTAC motif. (**B**) Yeast one-hybrid detected interactions between the *Sm*SPL6 and the promoters of *Sm4CL9* and *SmCYP98A14*. The p53HIS2/pGADT7-p53 and p53HIS2/pGADT7 served as positive and negative controls, respectively. (**C**) Schematic diagram of constructs used in assays of transient transcriptional activity. (**D**) *Sm*SPL6 activates the expression of *Sm4CL9* and *SmCYP98A14*. Effector *SmSPL6* was co-transformed with *p4CL9*-LUC/*pCYP98A14*-LUC reporters. All data are the means of three biological replicates, with error bars indicating SD; * represents a significant difference at *p* < 0.05 compared with the control.

It was reported that SPLs can regulate the expression of target genes by directly binding to the GTAC motif of target genes [19]. We found that the GTAC motif existed in the promoter regions of *Sm4CL9* and *SmCYP98A14* (Figure 8A). A yeast one-hybrid (Y1H) assay was performed to examine the physical interactions between the *Sm*SPL6 and the promoter regions of *Sm4CL9* and *SmCYP98A14*. Our results indicated that *Sm*SPL6 could bind to the promoter regions of the two genes (Figure 8B). In addition, a dual-luciferase transient transcriptional assay was performed to investigate whether *Sm*SPL6 might activate/regulate the expressions of *SmCYP98A14* and *Sm4CL9*, with the results indicating that it did (Figure 8D). These findings confirmed that *Sm*SPL6 binds directly to and activates the promoters of *SmCYP98A14* and *Sm4CL9* to promote the biosynthesis of RA and SalB.

## 3. Discussion

### 3.1. Function of SmSPL6 in Phenolic Acid Biosynthesis

Phenolic acids are an intense area of research in the secondary metabolism of *S. miltiorrhiza*. Previous reports have shown that several elicitors influence the production of phenolic acids [34]. These elicitors can be divided into two groups (biotic and abiotic), with the former containing both pathogenic and plant cell components [35,36], and the latter including Ag^+^ [37], MeJA [6], SA [38], etc. Elicitors can affect phenolic acid compounds via transcription factors, which activate or repress the expression of enzyme genes that are engaged in the phenolic acid biosynthetic pathway. It was reported that some MeJA-responsive transcription factors, such as *Sm*MYB97 [39], *Sm*MYB1 [40], *Sm*MYB111 [17], *Sm*bHLH148 [41], and *Sm*ERF1L1 [42], regulated the biosynthesis of phenolic acids via binding directly to and activating the promoters of enzyme-encoding genes involved in the biosynthetic pathway. For instance, *Sm*MYB1 positively regulates the biosynthesis of SalB by directly activating the key enzyme gene *SmCYP98A14* [40].

We found that *SmSPL6* responded to the treatment of exogenous MeJA (Figure 1B). Besides *AtSPL9*, the homeotic gene of *SmSPL6* is involved in the regulation of secondary metabolites [35]. We speculated that *Sm*SPL6 could be a candidate transcription factor for regulating the accumulation of phenolic acids in *S*. *miltiorrhiza*. To investigate whether *Sm*SPL6 mediates the biosynthesis of phenolic acid, we generated *SmSPL6*-overexpressed transgenic *S*. *miltiorrhiza* plants. The concentrations of RA and SalB were significantly increased in the transgenic lines. Y1H and dual-luciferase assays indicated that *Sm*SPL6 could bind to the promoters of the key enzyme genes *SmCYP98A14* and *Sm4CL9* and activate their expression (Figure 8). In this research, our findings demonstrated that *Sm*SPL6 was responsible for the generation of phenolic acid by directly activating the transcription of *SmCYP98A14* and *Sm4CL9* in *S*. *miltiorrhiza*.

### 3.2. Function of SmSPL6 in Anthocyanin Biosynthesis

Flavonoid-type anthocyanin is a critical type of secondary metabolite that can control plant fertility and protect plants from environmental stresses [43,44,45]. It is also beneficial for human health due to its antioxidant, anti-cancer activities and anti-inflammatory function [46,47]. Previous studies indicated that *At*SPL9 negatively regulates anthocyanin biosynthesis by disrupting the stability of the MYB-bHLH-WD40 transcription complex [33]. Meanwhile, *At*SPL9 directly regulates the expression of *DFR* (the key enzyme gene for anthocyanin biosynthetic pathway) to influence the metabolism of anthocyanin [33]. We detected the content of anthocyanin in the *SmSPL6*-OE transgenic lines and the control. Our results showed that overexpressed *SmSPL6* reduced the accumulation of anthocyanin in *S*. *miltiorrhiza* (Figure 5), which was consistent with the function of *At*SPL9 in regulating the production of anthocyanin. The expression levels of enzyme-encoding genes for the anthocyanin biosynthesis pathway were all down-regulated in the *SmSPL6*-OE lines (Figure 5C). We analyzed the promoter regions of those genes and found that the GTAC motif existed in the promoter regions of *CHS*, *F3H*, *F3′H*, *DFR*, and *ANS* (data not shown). Whether *Sm*SPL6 regulates the expression of these genes by directly binding to the GTAC motif will be investigated in our future studies.

Water-soluble phenolic acids and anthocyanin share a common phenylpropanoid pathway. Previous literature indicated that the positive regulators of phenolic acids may have different roles to play in the production of anthocyanin. For instance, overexpressed *SmMYB1* significantly promoted the accumulation of anthocyanin [40], while overexpressed *SmbHLH51* did not significantly alter anthocyanin generation [48]. Our results indicated that *Sm*SPL6 was a positive regulator for phenolic acids, but a negative regulator for anthocyanin, revealing that the regulatory mechanisms of secondary metabolites in plants is quite complex.

### 3.3. Function of SmSPL6 in Root Development

Root systems are essential for plant growth and survival due to their critical roles in the acquisition of water and nutrients. As is well known, the dried roots of *S*. *miltiorrhiza* are used as a traditional Chinese medicine; thus, improving the biomass and quality of roots is an important goal for the breeding of *S. miltiorrhiza*. Earlier reports have shown that *At*SPL9 and *At*SPL10 repressed lateral root growth in *Arabidopsis* [27]; 10-day-old *pSPL9:rSPL9* seedings exhibited fewer lateral roots than the wild type, whereas *pSPL10:rSPL10* seedings exhibited the delayed generation of lateral roots in contrast to *pSPL9:rSPL9*, which indicated that *At*SPL10 played a major role in lateral root growth [49]. We observed obvious changes in the root phenotypes, including fewer lateral roots, longer root lengths, and wider root diameters in the *SmSPL6*-OE lines (Figure 4C and Table 2). Although the root biomass decreased in the *SmSPL6*-OE lines, the phenotype of fewer lateral roots and longer root lengths are preferred for this traditional Chinese medicinal material.

The plant hormone auxin plays vital roles in the growth and development of roots [50,51]. Whether *Sm*SPL6 inhibits lateral root development by regulating the levels of endogenous auxin should be further investigated for *S. miltiorrhiza*. In *Arabidopsis*, the expression of *AtSPL9* and *AtSPL10* was induced through the treatment of exogenous IAA [49]. Our data indicated that *SmSPL6* was responsive to auxin; however, its expression was inhibited by the exogenous IAA treatment (Figure 1B). The opposite expression responses of *SmSPL6* and *AtSPL9* to IAA may have been due to the application of different concentrations of exogenous IAA. In the present study, 100 μM IAA was used to spray the *S. miltiorrhiza* seedlings, while the *Arabidopsis* seedlings were treated with 10 μM IAA. Whether *SmSPL6* is induced by low concentrations of IAA will be further investigated.

Collectively, these results elucidated the role of *SmSPL6* in the regulation of secondary metabolites and lateral root development in *S. miltiorrhiza. *The functional consistency of *Sm*SPL6 and *At*SPL9 for inhibiting lateral root development and the biosynthesis of anthocyanin revealed the conservatism of the SPL family in plants, while the function of *Sm*SPL6 in promoting the generation of SalB demonstrated the species specificity of SPL members. In the following research, we will try to generate *SPL6* mutant lines in *S. miltiorrhiza* using the CRISPR/Cas9 system to better elucidate the function of *Sm*SPL6 transcription factor.

## 4. Materials and Methods

### 4.1. Plant Materials and Hormone Treatments

*S. miltiorrhiza* seeds (Shangluo country, Shaanxi province) were sterilized and cultured on Murashige and Skoog basal medium for the transformation experiments, as described by Yan and Wang [52]. *Arabidopsis thaliana* ecotype Columbia-0 and tobacco (*Nicotiana tabacum*) were cultivated in a growth chamber at 22 °C under a 16 h light:8 h dark photoperiod.

Stems, leaves, primary roots, lateral roots, pistil, stamen, corolla, and calyx were separately collected from 2-year-old *S. miltiorrhiza* plants at the flowering stage for RNA extraction in an experimental field at Shaanxi Normal University.

Two-month-old *S. miltiorrhiza* plantlets were treated with 0.1 mM IAA, 0.1 mM GA_3_, 5 mM MeJA, or 0.1 mM ABA as previously described [53], which were then collected for RNA extraction following treatment for 10 min, 30 min, 1 h, 2 h, 3 h, and 9 h, respectively.

### 4.2. Gene Cloning and Sequence Analysis

The promoter fragments and full-length cDNA of *SmSPL6* were amplified from the DNA and cDNA of the 2-month-old *S. miltiorrhiza* plantlets, respectively. The PCR products were inserted into pMD19-T (TaKaRa, Dalian, China) vector and confirmed by sequencing. The cis-elements in the promoter fragment were predicted by PlantCARE (http://bioinformatics.psb.ugent.be/webtools/plantcare/html/) (Accessed on 21 July 2021). All primers used in this study are listed in supplementary Table S1.

### 4.3. QRT-PCR

Total RNA was extracted using the Tissue Total RNA Isolation Kit (Vazyme, Nanjing, China) and reverse transcribed to cDNA using HiScript II Reverse Transcriptase (Vazyme, Nanjing, China). The qRT-PCR was performed using the SYBR green qPCR Mix (Vazyme, Nanjing, China) using a real-time fluorescence quantitative PCR detection system (Roche). *SmUbiquitin* served as an internal control. The expression levels of *SmSPL6* and other genes were calculated by the 2^−ΔΔCT^ analysis method [54].

### 4.4. Vector Construction and Genetic Transformation

To generate the overexpressed vector, the 1083 bp ORF of *SmSPL6* was inserted into the overexpression vector pEarlygate202 using the Gateway recombinatorial cloning system (Invitrogen, Carlsbad, CA, USA) [55]. The 862 bp promoter fragment of *SmSPL6* was cloned and inserted into the *Sal*I and *EcoR*I (TaKaRa, Beijing, China) sites of the pCAMBIA1391z vector to drive the expression of *GUS*. 

The *SmSPL6*-overexpressed genetic transformation of *S. miltiorrhiza* was achieved via an *Agrobacterium*-mediated method, which was established previously [52], and selected on an appropriate medium supplemented with 10 mg/L glufosinate-ammonium (Nalgene, United States). 

The transgenic *Arabidopsis* expressing *ProSmSPL6*::*GUS* was obtained via the *Agrobacterium*-mediated floral dip method [56]. Transgenic seeds were selected on agar media with 25 mg/L hygromycin (Roche, Switzerland).

### 4.5. β-Glucuronidase (GUS) Histochemical Staining

The T2-generation transgenic *Arabidopsis* expressing *ProSmSPL6:GUS* was used for GUS staining according to a previously described protocol [57].

### 4.6. Subcellular Localization of SmSPL6 Protein

To investigate the subcellular localization of the *Sm*SPL6 protein, *SmSPL6* was integrated into a pEarlygate103 vector via the Gateway recombinatorial cloning system (Invitrogen, Carlsbad, CA, USA) [55]. Next, the recombinant pEarlygate103-*SmSPL6* and pEarlygate103 vectors were transformed to onion epidermal cells, respectively, and the GFP fluorescence signals were observed as previously described [17].

### 4.7. Transcription Activation Assays

The ORF of *SmSPL6* was integrated into the pGBKT7 vector to fuse with the GAL4 DNA-binding domain (BD) gene. The recombinant material was then transferred into *Saccharomyces cerevisiae* strain AH109 (Weidi Biotechnology, Shanghai, China) via the lithium acetate-mediated method [58]. The transformants were grown on SD/-Trp medium (Coolaber, Beijing, China) at 29 °C for 2–3 days, and then screened on a SD/-Trp/-Ade/-His/X-α-gal yeast medium (Coolaber, Beijing, China) to assay the transactivation activity.

### 4.8. Y1H Assays

The ORF of *SmSPL6* was cloned into the *Sma*I and *Xho*I (TaKaRa, Beijing, China) sites of the pGADT7 vector. The 1146 bp promoter fragment of *SmCYP98A14* was inserted into the *Sma*I and *Mlu*I (TaKaRa, Beijing, China) sites of the pHIS vector to generate the recombinant pHIS2-*pCYP98A14*. The pHIS2-*p4CL9* was obtained in an earlier study [53]. The recombinant vector pairs were co-transformed into *Saccharomyces cerevisiae* strain Y187 (Weidi Biotechnology, Shanghai, China) via a lithium acetate mediated method [58]. The transformants were cultured on SD/-Trp/-Leu medium (Coolaber, Beijing, China) and then detected on SD/-Trp/-His/-Leu medium (Coolaber, Beijing, China), which was supplemented with 60 mM 3-amino-1,2,4-triazole (3-AT) (Coolaber, Beijing, China).

### 4.9. Dual-Luciferase Assay in Tobacco Leaves

The ORF of *SmSPL6* was inserted into the *Sma*I and *Xho*I (TaKaRa, Beijing, China) sites of pGreenII-62SK to obtain an effector vector. The promoter fragment of *SmCYP98A14* was cloned into the *Hin*dIII and *Bam*HI (TaKaRa, Beijing, China) sites of pGreenII 0800-LUC vector as a report vector. The recombinant vector of *pSm4CL9*-pGreenII 0800 LUC was synthesized as per our previous report [53]. The synthesized effector and reporter vectors were subsequently co-transferred into tobacco leaves via an *Agrobacterium*-mediated transformation. Three days following infiltration, the activities of LUC (firefly luciferase) and renilla luciferase (REN) were determined as described previously [59].

### 4.10. Determination of Active Compounds

Two-month-old *SmSPL6-*overexpressed transgenic *S. miltiorrhiza* plantlets and the control were used for anthocyanin and phenolic acid analysis. The content of anthocyanin was detected as previously described [60]. The SalB and RA concentrations were measured via the HPLC method. All separations were carried out at a constant temperature (30 °C) with the specific process and parameter settings being performed as previously described [17].

## Figures and Tables

**Table 1:** Cis-elements analysis of *SmSPL6* promoter.

Cis-Elements	Sequence	Number	Functions
ABRE	ACGTG	1	abscisic acid responsiveness element
Box4	ATTAAT	3	involved in light responsiveness
Box II	TGGTAATAA	1	part of a light responsive element
CAT-box	GCCACT	1	related to meristem expression
G-box	CACGTC	1	involved in light responsiveness
P-box	CCTTTTG	1	gibberellin-responsive element
I-box	CCTTATCCT	1	part of a light responsive element
TGA-element	AACGAC	1	auxin-responsive element

**Table 2:** Root parameters of the *SmSPL6* overexpressed (OEs) transgenic lines and the control.

Lines	Roots Length/cm	Fresh Weight/g	Lateral Roots Number
control	8.54 ± 0.87 ^c^	0.760 ± 0.082 ^a^	>20 ^a^
OE5	16.02 ± 0.52 ^b^	0.625 ± 0.064 ^a^	15 ± 2 ^b^
OE6	17.15 ± 0.49 ^b^	0.600 ± 0.028 ^a^	15 ± 2 ^b^
OE8	20.10 ± 0.71 ^a^	0.385 ± 0.035 ^b^	12 ± 2 ^b^

Note: One-way ANOVA (followed by Tukey’s comparisons) tested for significant differences between means (indicated by different letters at *p* < 0.05).

## Data Availability

Not applicable.

## Supplementary Materials

The following are available online at https://www.mdpi.com/article/10.3390/ijms22157895/s1, Table S1: List of primers used for PCR and qRT-PCR.
