# Supplementary material for: SmSPL6 Induces Phenolic Acid Biosynthesis and Affects Root Development in Salvia miltiorrhiza
Source: Int J Mol Sci. 2021 Jul 23;22(15):7895. doi: 10.3390/ijms22157895 (PMC8348295; doi:10.3390/ijms22157895)
Supplement: Supplementary file 1 [file ijms-22-07895-s001.zip › ijms-1291112-supplementary.pdf]

Supplementary Material

Table S1. List of primers used in the study.

Primer	Oligo Sequence 5′ to 3′
RT-*SmUbiquitin*-F	ACCCTCACGGGGAAGACCATC
RT-*SmUbiquitin*-R	ACCACGGAGACGGAGGACAAG
SmSPL6-F	ATGGATAAGGGCTCATCTTCC
SmSPL6-R	TCAAAGTGACCAATTGATATGCT
ProSmSPL6-F	GTTTTGTTGAAGAATGATGAAGTGA
ProSmSPL6-R	AGAACCCATTCATTGCTGCTC
RT-*SmSPL6*-F	TCGGAACGAGTAATGGGAAAT
RT-*SmSPL6*-R	TCTTGAGCCCCAGGACTGAG
RT-*CHS*-F	ATCACCAACAGCGAGCATAAGAC
RT-*CHS*-R	ATCTTCGACTTGGGCTGCC
RT-*F3H*-F	CATCTACTCCAACATCGGACAGC
RT-*F3H*-R	CCCCACATAAGGTTCATCAACAG
RT-*F3′H*-F	TCAGGCTTTGAGCAATGGGAAGT
RT-*F3′H*-R	TGAGTCGCATGGGCAGAGGAA
RT-*DFR*-F	CTCACTACTCCATCATAAAGCAAGG
RT-*DFR*-R	AGTAGAAAACGGCAGCAATCCT
RT-*ANS*-F	GGCAAATGGGTCACCGCAAAA
RT-*ANS*-R	GGCTCGCAGAAAACTGCCCAAG
RT-*TAT1*-F	CAACTGCTGGTCTTCCACAAAC
RT-*TAT1*-R	GCGAGCCAAAACGGACA
RT-*TAT2*-F	CGGAGATCCATCCGCCTTCC
RT-*TAT2*-R	CGGGAATACCGACGGTGGAG
RT-*TAT3*-F	TGCTGAAACTGCCAAGAGGCT
RT-*TAT3*-R	CCGGGCACCAACCATCTCTT
RT-*HPPR1*-F	TGACTCCAGAAACAACCCACATT
RT-*HPPR1*-R	CCCAGACGACCCTCCACAAG
RT-*HPPR3*-F	GCGCTACCGCTCCTTGAGAT
RT-*HPPR3*-R	CACAAATCCGCCGCGAAGTC
RT-*PAL1*-F	GATAGCGGAGTGCAGGTCGTAC
RT-*PAL1*-R	CGAACTAGCAGATTGGCAGAGG
RT-*PAL3*-F	CTCCACCCGTCGAGGTTCTG
RT-*PAL3*-R	TCTGCATGAGCGGGTACGTG
RT-*C4H1*-F	CCAGGAGTCCAAATAACAGAGCCG
RT-*C4H1*-R	GCCACCAAGCGTTCACCAAGAT
RT-*4CL1*-F	TCACCCATGCCGGATTCGAG
RT-*4CL1*-R	AGATCGCGCCGATGAAGGAG
RT-*4CL2*-F	GCGGCGTAGTGCTTCACCTTT
RT-*4CL2*-R	TCGCCAAATACGACCTTTCC
RT-*4CL9*-F	AAAGCACCGCCGTAAGTG
RT-*4CL9*-R	AGCCCAGTTGCAGAATCAAC
RT-*RAS1*-F	CCAAAGTCAATTATGCCAAGGG
RT-*RAS1*-R	GTCGGATAGGTGGTGCTCGT
RT-*RAS2*-F	ACTCGGTTCAAATGCGGTAG
RT-*RAS2*-R	GGGCTGGTATTCGTCGTG
RT-*RAS5*-F	GGGAGTGTTGTTCGTGGAGG
RT-*RAS5*-R	GCAAAAGTGGGAAGGTGGAA
RT-*CYP98A14*-F	ACGTGCGTGTTGCTACGAGAC
RT-*CYP98A14*-R	CGTCGCCAGTGCTGCAACTAA
